# Critical Care Teamwork in the Future: The Role of TeamSTEPPS^®^ in the COVID-19 Pandemic and Implications for the Future

**DOI:** 10.3390/healthcare11040599

**Published:** 2023-02-17

**Authors:** Carol A. Terregino, Sugeet Jagpal, Payal Parikh, Archana Pradhan, Paul Weber, Lauren Michaels, Olivia Nicastro, Jared Escobar, Hanin Rashid

**Affiliations:** 1 Rutgers Robert Wood Johnson Medical School, Rutgers University, Piscataway, NJ 08854, USA; 2 Robert Wood Johnson Barnabas Health, New Brunswick, NJ 08901, USA

**Keywords:** TeamSTEPPS^®^, teamwork, communication, COVID-19 pandemic, critical care

## Abstract

At our institution, we observed inconsistency in the application of structural facilitators for interprofessional teamwork such as handoffs and communication of contingency planning, complete formation and engagement of teams on interprofessional rounds, regular situation monitoring, interprofessional huddles, use of “check back” during code situations, and standard debriefings after codes and procedures (TeamSTEPPS^®^). To enhance team performance, we piloted TeamSTEPPS^®^ training and reinforcement for all healthcare team members in the medical intensive care unit (MICU), inclusive of trainees, advanced practice providers (APPs), nurses, and respiratory therapists rotating through the unit. Seven months after the training launch, the initial COVID-19 surge interrupted the reinforcement stage of the pilot providing an opportunity to study the retention of TeamSTEPPS^®^ principles and its potential role in response to a crisis. We conducted interprofessional focus groups after a year of crisis management during the pandemic. Themes revealed how TeamSTEPPS^®^ training impacted teamwork and communication, as well as factors that influenced the use of TeamSTEPPS^®^. This work points to the value of team training in unexpected scenarios. Additional studies at multiple sites are needed to determine scalability for all MICU teams or for onboarding new team members.

## 1. Introduction

In military and aviation settings, training and deliberate practice are sine qua non for daily operations, continuous improvement, and crisis management. Recent healthcare advances have advocated for teamwork and leadership training. There is clear face value for teamwork training in healthcare with studies reporting variable outcomes related to attitudes and behaviors [1]. The literature also indicates that more rigorous study of the effects of teamwork training including longitudinal research on retention and performance during crises is needed [2].

The presence of interprofessional staff, such as in the medical intensive care unit (MICU), does not necessarily lead to high-performing teams without deliberate thought on training and facilitation. A previous qualitative study on the perceptions of interprofessional collaboration revealed the need to develop both structural (clinical protocols, checklists, daily rounds, and information technology) and cultural (accessibility, trust, value, and leadership) facilitators for higher performance teams [3].

At our institution, we observed inconsistency in the application of structural facilitators such as handoffs and communication of contingency planning, complete formation and engagement of teams on interprofessional rounds, regular situation monitoring, interprofessional huddles, use of “check back”(the verification of verbally communicated information during emergent situations such as cardiac arrest), and standard debriefings after emergent situations and procedures in our ICU. Additionally, we noted the absence of cultural facilitators such as trust for interprofessional collaboration [3]. The need to deliberately train teamwork facilitators was evident.

To enhance team performance, we piloted TeamSTEPPS^®^ [4] (Team Strategies to Enhance Performance and Patient Safety, an Agency for Healthcare Research and Quality program) training and reinforcement for all healthcare team members in the MICU, inclusive of physician trainees, advanced practice providers (APPs), nurses, and respiratory therapists. The program is designed to enhance learner teamwork behaviors through five evidence-based teamwork competencies: proper team structure, communication, leadership, situation monitoring (active assessment of elements affecting the situation in which the team functions), and mutual support (assisting, providing feedback, supporting, and advocating) [5,6]. Application of the TeamSTEPPS^®^ model to team-based environments has been shown to increase teamwork attitudes (mutual respect and confidence in team capabilities), skills (team structuring, communication, leadership, situation monitoring, and mutual support), team performance (efficiency, productivity, accuracy, and adaptability), and patient safety. TeamSTEPPS^®^ has demonstrated effectiveness and positive teamwork training outcomes in over 200 publications across various specialties and healthcare professions [5,7,8,9,10]. TeamSTEPPS^®^ is among several tools used to improve teamwork including Crew Resource Management [1,2,11] and High-Reliability Organization [12,13].

Seven months after the training launch, the initial COVID-19 surge interrupted the reinforcement stage of the pilot. This provided the opportunity to observe and assess the natural time course for retention of TeamSTEPPS^®^ principles after initial training and the potential role of these principles in response to crisis situations. This study presents the qualitative evaluation of how the initial training affected performance during the pandemic and what aspects of the training were retained.

## 2. Materials and Methods

### 2.1. Research Team

The research team consists of medical educators, academic physicians with expertise in critical care medicine, patient safety, and quality improvement, a critical care nursing director, a director for the APP fellowship, a veteran medical student, and an educational psychologist with expertise in qualitative analysis. Specifically, Dr. Terregino is the senior associate dean for education and academic affairs experienced in undergraduate medical education and transition to residency. She is a TeamSTEPPS^®^ master trainer and along with fourth-year medical student Jared Escobar, Dr. Terregino conducted the didactic sessions and transcribed all of the focus groups. Dr. Weber directs the undergraduate medical education curricular thread, Health Systems Science, and is also a TeamSTEPPS^®^ master trainer. His role was direct observation of teams. Dr. Archana Pradhan is the associate dean for clinical education and a TeamSTEPPS^®^ master trainer. Her expertise in design thinking enhanced the training of staff. Dr. Jagpal is the director of quality in the MICU and Dr. Parikh is the vice chair for quality and safety in the Department of Medicine. They were boots on the ground as “on the job” trainers and reinforcers of TeamSTEPPS^®^ training methodology. Lauren Michaels directs nursing in the MICU and Olivia Nicastro leads the APPs. Dr. Rashid provided expertise in preparing the focus group questions and facilitating qualitative analysis.

### 2.2. Initial Instruction

There exists a standard undergraduate medical education (UME) curriculum on teamwork, delivered within the context of high-functioning military teams, modified from TeamSTEPPS^®^ and implemented by Rutgers Robert Wood Johnson Medical School students who are veterans, and education faculty [14]. The principal author (CT) and a former US Marine medical student (JE), both TeamSTEPPS^®^ master trainers, revised the training for interprofessional practitioners, residents, fellows, attendings, and APPs and engaged them in discussions of their MICU experiences. They applied TeamSTEPPS^®^ tools to case scenarios and received a trifold pocket card with TeamSTEPPS^®^ tools for ease of reference. Participants also attended didactic sessions on quality and safety in healthcare. We also distributed a trifold educational tool to all critical care nurses, respiratory therapists, pharmacists, and social workers in an “on the job” training model.

Two study team members (PP and SJ), both with a background in quality and safety principles, each spent one half-day a week observing team functioning while rounding with the teams, during handoffs, and during other critical events. They reinforced interprofessional work-place specific teamwork by encouraging team members to identify barriers to optimal patient care and empowering them to conceive solutions that may lead to sustainable improvements. Additionally, they conducted biweekly huddles with residents, fellows, nursing leadership (LM), staff nurses, and critical care advanced practice providers (ON). The other study team members (AP, PW, CT), employed design thinking processes and served as bridges to drive the systems improvement needs identified during the huddles. The reinforcement stage was halted at the onset of the March 2020 COVID-19 surge. This interruption provided an opportunity to study the retention of TeamSTEPPS^®^ principles in response to a crisis.

### 2.3. Participants

In April 2021, we identified all internal medicine residents, critical care fellows, critical care attending physicians, APPs, nurses, and respiratory therapists, who participated in our original TeamSTEPPS^®^ training and reinforcement stages and who practiced in the MICU at RWJBarnabas Health Robert Wood Johnson University Hospital (RWJUH) during the COVID-19 surge. All participants were recruited by SJ. Participants were approached individually.

### 2.4. Study Design

We invited participants to one of five focus groups where we evaluated how the initial training affected performance during the pandemic and what aspects of training had been retained.

Five focus groups were conducted on the Rutgers Office of Information Technology’s licensed Zoom virtual platform by the principal investigator and moderator (CT) to guarantee uniformity. To ensure consistency between the focus groups, a semi-structured interview was developed and followed closely (See Appendix A for interview protocol). The moderator encouraged all participants to express their thoughts openly and emphasized the need for differing opinions to capture potentially varying experiences. To create an unbiased atmosphere, and preserve anonymity, the security function on Zoom was used to prevent participants from turning on their cameras and to block the participant list from being visible. Degree, e.g., RN, MD, etc., was the only visible identifier and the recording function was voice only. There were five focus groups lasting approximately 90 min. The moderator also transcribed the interviews.

### 2.5. Qualitative Analysis

We analyzed our data using Braun and Clarke’s six-phase approach to thematic analysis [15,16]. We used an inductive approach, therefore coding the data without a pre-existing theme and allowing for analysis to be data-driven. First, all investigators initially read through the transcripts and provided initial ideas for codes. Next, the principal investigator (CT) and an investigator trained in qualitative methods (HR) systematically reviewed the data and generated initial codes guided by the study’s aim. Any statements mentioning initial TeamSTEPPS training or its use and how it impacted their work were coded. Codes were then confirmed by the support of the re-emergence of those same codes between the initial coders as well as the presence of the same codes across several transcripts. All codes were discussed between the two initial coders. Codes were then categorized into concepts and patterns to represent themes that helped interpret the codes and address the research question. When saturation was reached (i.e., no new codes or themes were identified), an initial codebook was created. In order to assure reliability, all remaining investigators coded two transcripts using the codebook. HR served as the third coder for each of the transcripts to ensure inter-rater reliability and then resolved ambiguity/disagreement through discussion with the principal investigator. After review by all authors, the themes were further refined. HR and CT then finalized the codebook (Appendix B) with clear names of themes and codes, definitions, and sample excerpts. ATLAS Ti software was used for tracking final coding. Finally, all findings were reviewed by all investigators following the report of all coded material.

### 2.6. Ethics

The study was conducted after approval by the Rutgers eIRB (#pro2020003187). All participants provided informed consent. Respondents received a $10.00 Starbucks gift card as an incentive for participation.

## 3. Results

Twenty-seven participants (nine physician residents or fellows, five attending physicians, seven nurses, three advanced practice providers, and three respiratory therapists) attended one of five focus groups. We identified three total themes and 19 total codes: (a) how TeamSTEPPS impacted teamwork (6 codes), (b) how TeamSTEPPS impacted communication (5 codes), and (c) factors that influenced the use of TeamSTEPPS during a crisis (8 codes).

### 3.1. How TeamSTEPPS^®^ Impacted Teamwork

Participants commented on feeling a change in the culture of the interprofessional team for the care of their patients. Within this theme, we identified six codes, whole, role, trust, mental model, consistency/efficacy, and empathy, as described below.

#### 3.1.1. Mental Model (31 Quotes)

The mental model was the most quoted code and was reported in all five focus groups. TeamSTEPPS^®^ requires establishing a shared mental model to provide team members with a common understanding of who is responsible for each task in order to ensure that the whole team is on the same page.

A nurse explained that a shared mental model improved interdisciplinary teamwork through communication, stating, “…having that constant communication, the giving and receiving of information, so that we all are on the same page as far as the patient care and the plan for the day and the physician group checking back to make sure what we discussed in the morning either happened or didn’t happen and we have to shift and make another plan basically the constant communication among the disciplines to be on the same page”. Having a shared mental model allows for synchronicity to provide quality care.

As a doctor explained, the team’s common goal was patient care. They explained, “discussing as a team about the patient’s prognosis and getting on the same page in that way. I think that it’s not just the physician’s perspective that’s important there. I think everyone’s input is important and should be valued and once everyone’s on the same page, I think it makes patient care much easier going forward”.

#### 3.1.2. Whole (23 Quotes)

Participants in all the focus groups reported identifying a broadened definition of who the “whole” team is and who should be included in rounds at the beginning of a shift. An MD stated, “I think, each time we round now we make sure the nurses are here for rounds as well as the pharmacist. I feel like since we had the training, we make sure that each time we round, we have the whole team here to make sure everyone knows the plan for the rest of the day”. Nurses and respiratory therapists indicated that they enjoyed being included and made to feel validated and credited as part of the team. A respiratory therapist highlighted this point by stating, “maybe there was something going on that needed to be settled before the actual rounds could start and by acknowledging all team members you sort of give validity and credit to the people that are participating…I was brought into the group and made to feel like I was an active participant”. Overall comments reflected how TeamSTEPPS^®^ training encouraged greater inclusion of the whole team which seemed positive for all members of the team.

#### 3.1.3. Trust (20 Quotes)

Participants in four of the focus groups cited an increase in trust amongst the team, including improved rapport and respect. Dialogue between a nurse and respiratory tech highlights how TeamSTEPPS^®^ training helped create greater trust amongst the team as a product of the briefing process.

RN: I feel like it definitely has increased the amount of trust we have in each other; if you make suggestions based on your experience and your rapport with them, I believe they are willing to take a lot more of your suggestions into consideration and carry it out. 

RRT: Yes; you build a rapport with the doctors and nurses, and you gain trust.

#### 3.1.4. Role (17 Quotes)

Trainees highlighted the importance of everyone knowing their role so they could meaningfully contribute to the team in all five focus groups. A nurse explained, “I do recall the importance of the leader of the teams to kind of make sure everyone knows, their role and to contribute in a meaningful way in order for the teams to function smoothly”. A doctor underscored the benefits of knowing their role to make sure the team functions better: “…residents change frequently and some of them haven’t had any training. It wasn’t as organized and now we make sure that one person times, one person writes down the drugs, like we don’t do duplicate jobs”.

When discussing how these defined roles improved teamwork, an AAP stated, “Personally, I think that it actually impacted it in a positive way. Discussing it with the attending and nurses and they said that if something happens, this would be this person’s role. This person will make sure that to bring the equipment and all of that”.

#### 3.1.5. Consistency/Efficiency (16 Quotes)

TeamSTEPPS^®^ is meant to promote consistency and efficiency in how things are completed so that the team can be more efficient in providing patient care. Participants across the five focus groups discussed this impact. For instance, a physician explained, “It makes it more efficient because let’s say the team doesn’t communicate enough and leaves a bunch of orders and the nurse is completely unaware of the plan. The team walks away, and the nurse has to then find the resident or the team member to try to find out what exactly was the plan. It’s highly inefficient if it leads to multiple phone calls or conversations which we could’ve accomplished, while on rounds”.

#### 3.1.6. Empathy (16 Quotes)

As a result of increased teamwork, participants in all five focus groups reported increased empathy toward team members. A nurse highlighted that knowing what everyone is doing and going through helped promote empathy. “I definitely try to be more mindful of what some of the other team members might be going through when I find myself getting frustrated”. As a physician noted, “I actually think there is even more camaraderie between the nurses and the residents”.

### 3.2. How TeamSTEPPS^®^ Impacted Communication

Participants indicated that TeamSTEPPS improved communication between the team and with their supervisors. Themes indicated a raised awareness of communication issues and highlighted the importance of good communication. Within this theme, we identified five codes: situation monitoring, briefing/debriefing, safety, transition of care, and units.

#### 3.2.1. Situation Monitoring (26 Quotes)

TeamSTEPPS^®^ defines situation monitoring as a way for the team to provide awareness of what is going on and enable individuals to adapt to changes in a situation. Checking in on team members, the environment, and patient progress were examples of engaging in situation monitoring, which was moderated by communication of new information with other team members. Participants from all focus groups discussed engaging in situation monitoring.

An RN summarized, “I guess that’s the best way to put it, just more frequent questions, ‘Are you Okay’, ‘Do you need more help’ or ‘anything else we can do for you?’” An RN from a different focus group elaborated on how situation monitoring improved communication and ultimately improved patient care. “Because we’re constantly looking at all their systems and making sure that everything is flowing and working the way it’s supposed to. Sometimes it’s working, sometimes it isn’t and that’s also important too, because we need to know if the patient is heading towards let’s say renal failure or whatever”.

#### 3.2.2. Briefing/Debriefing (22 Quotes)

Two of the most effective strategies of TeamSTEPPS are engaging in a team brief to share the plan for the day and a debrief to review performance and discuss critical events at the end of the day. Participants from all focus groups reported still engaging in some level of briefings and debriefings.

MODERATOR: “Do you think there’s a change compared to before the training?”

RN: “We’re doing that all the time now”.

They also highlighted how briefs and debriefs impacted communication. An MD elaborated:

“So, one thing that I took away from the training was surrounding family meetings and communicating with families. Oftentimes before TeamSTEPPS we would pretty much set up a meeting with a family, go in and not really have an extended conversation before the meeting. So, what I’ve started doing, this one thing we did do during COVID-19, we would talk in advance about our goal for the meeting what we were going to tell the family, anticipate different responses and how we would react to that and respond. We would have the meeting, and then we did do a debriefing after our family meetings, and I think that that was extremely helpful and made our family meetings, more efficient and more productive”.

#### 3.2.3. Safety (18 Quotes)

Participants from all focus groups reported patient safety as an important product of improved communication as a result of TeamSTEPPS^®^ training. An RN stated, “As an experienced nurse, I realized that communication is key to our roles with each other and for the safety of our patients and ourselves…” Moreover, an APP explained that the improved communication was key for patient safety during COVID-19: “…the ultimate goal during COVID-19 was to get them out of the unit safely. I think the key was communication… I think our plan at the end is that we care about patient safety; it is what we want all the time”.

#### 3.2.4. Transition of Care (16 Quotes)

Participants reported better communication for the transition of care during sign-outs, including more comprehensive discussions of significant events and care priorities. All focus groups discussed better communication during the transition of care after TeamSTEPPS^®^ training.

An MD explained, “I think the other change that’s been made is that the evening attending intensivist is also supposed to be rounding with the resident who’s on for the night, so I think that also makes a difference and makes the residents and team feel more prepared if there are changes that occur with patients’ clinical status”.

#### 3.2.5. Units (15 Quotes)

TeamSTEPPS^®^ emphasizes the importance of communication between hospital units. Participants from four groups reported the use of TeamSTEPPS^®^ communication tools to assist in work between hospital units. An MD summarized the findings by explaining, “I think that providing more generalized structure and maybe standardizing communication amongst all interdisciplinary care providers in other units would be helpul. I can’t imagine it would be anything other than improved”.

### 3.3. Factors That Influenced the Use of TeamSTEPPS^®^ Strategies

Participants indicated different factors that supported or inhibited the use of TeamSTEPPS^®^ strategies. The initial TeamSTEPPS^®^ training, the COVID-19 surge, and the MICU environment all presented both positive and negative examples of the practicality of TeamSTEPPS^®^ functionality. In addition, patient acuity and hierarchy within the hospital was a consistently cited barrier to the use of TeamSTEPPS^®^ strategies.

#### 3.3.1. Perception of Training Perceived by Trainees (49 Quotes)

Participants from all focus groups discussed their perceptions of the actual TeamSTEPPS^®^ training process and reinforcement of that training, and how the training impacted their use of TeamSTEPPS^®^ strategies in both positive and negative ways.

##### Positive

Participants discussed that the training had a positive impact on their overall care. Specifically, one MD discussed how the training had a positive influence on the team: “In terms of functioning as a team, probably because of the training, I think we approached it as a team, and that we were able to get through it as a team and without everyone’s individual roles, I think we would have been much worse off”. An RN in another focus group stated, “I think the most memorable part of the training is just the willingness of the team to reach out and always find better ways to do things. I think it’s important that there’s always innovation in providing collaborative care and a willingness to participate in what is better”.

##### Negative

Some participants detailed a lack of follow-up to the initial TeamSTEPPS^®^ training as a barrier to the use of TeamSTEPPS^®^ strategies. For instance, an MD stated it was difficult to know if TeamSTEPPS^®^ was being properly followed. “Nobody’s telling you if you’re doing something right or wrong. I think ongoing feedback. It doesn’t even have to be a session, but just ongoing reminders you should use TeamSTEPPS^®^ or just checking”. An MD from another focus group stated that with new residents joining and without continuous training, it can be difficult to maintain the program. “The units became very diversified. I think there were also different physicians involved and I’m not sure if they received training”.

#### 3.3.2. MICU (26 Quotes)

Participants reported that the MICU environment itself influenced the team’s ability to utilize TeamSTEPPS^®^ strategies. Although some mentioned that they felt the MICU environment enhanced their ability to use TeamSTEPPS^®^ strategies, others felt like it equally challenged TeamSTEPPS^®^ utilization.

##### MICU Environment Positive (13 Quotes)

Four of the five focus groups mentioned that team STEPPS was positive for the MICU environment and the environment itself enhanced their ability to employ TeamSTEPPS^®^ strategies. As an RN explained, the MICU environment requires teamwork: “I think this was always, in my opinion, the climate of MICU. We have the same goals, and that is to get our patients better, so that is the pulse of the MICU.”

The smaller sized teams in the MICU were repeatedly cited as a benefit for TeamSTEPPS^®^: “The MICU provides a really nice benefit in that the nurses and physicians are constant, the attendings and the fellows- even though there’s a change with residents- the APNs are constant. It’s a very small pool that you can target, and it’s so much easier to do this and see differences from TeamSTEPPS^®^”.

##### MICU Environment Negative (13 Quotes)

Three of the five focus groups discussed the MICU environment as challenging for adherence to TeamSTEPPS^®^. One common complaint was the rapidly changing teams of the MICU. There are multiple reasons why teams change so rapidly in the MICU. The most fluid aspect of critical care teams is the four-week rotation of residents in addition to changes in attending intensivist coverage which may occur weekly. There may be night float residents and attending coverage. Additionally, nurses staff the MICU in shifts and may not work consecutive days. Consultants may be called upon for the MICU and they may not have TeamSTEPPS^®^ training. These dynamics impair the ability to regularly train and reinforce teamwork behavior. Similarly, such strategies become even more critical, ensuring appropriate communication between rapidly changing team members.

As a physician explained, “Part of the issue in the MICU is that plans change very rapidly. You’re trying to balance actually doing patient care versus communicating with multiple different providers that are taking care of the patient. That’s what makes it difficult to constantly be updated on everything that’s going on with everyone”. Others explained that since different specialties interact and influence the MICU team, not all members were TeamSTEPPS^®^ trained therefore complicating compliance.

#### 3.3.3. COVID-19 (19 Quotes)

Participants discussed the benefits of using TeamSTEPPS^®^ during the COVID-19 pandemic while others mentioned how the COVID-19 surge complicated the use of TeamSTEPPS^®^ strategies.

##### COVID-19 Positive (11 Quotes)

Participants from four of the five focus groups mentioned that TeamSTEPPS^®^ was useful for maintaining structure during the pandemic. When asked about their use of TeamSTEPPS^®^ strategies during COVID-19, an RN stated, “I would say it definitely has improved, even more so after the COVID-19 surge. I feel like the rounds are a little bit longer, a little more focused on plan of care and how do we move forward”.

Another RN discussed how TeamSTEPPS^®^ and COVID-19 together may have contributed to increased teamwork. “I was saying that I think we do take care for each other, but I don’t know if that’s because of the training or just because of COVID-19. Because of the surge and everything we’re forced to work together and try to take care of these patients who are dying left, right and center. So, I think that I feel more of a stronger connection with the rest of the team”.

##### COVID-19 Negative (8 Quotes)

Participants from four of five focus groups indicated that COVID-19 made utilizing TeamSTEPPS^®^ more difficult. As an MD explained, it was difficult to keep up with TeamSTEPPS^®^ strategies when there was so much urgency. “I think everybody understood and appreciated the training, but after that training happens, you know we all apply things as we saw fit and maybe the pandemic showing up played a big role in this, because everything was kind of thrown up in the air, so it was more going into reactive mode for several months”.

#### 3.3.4. Hierarchy (13 Quotes)

Four of the five groups reported power dynamics leading to an inhibition of honest conversation and limited the ability of team members to appropriately adopt their TeamSTEPPS^®^ role. An MD explained that he noticed the team was not comfortable using the dialogue required. “There’s a level of discomfort. There could be information that they are not sharing because of fears it may affect team dynamics”. A nurse elaborated on this point stating that “TeamSTEPPS^®^ dialogue can come off as authoritative and this was uncomfortable”.

#### 3.3.5. Acuity (8 Quotes)

Participants from four of the five focus groups stated that the critical status of patients impacted their ability to engage in TeamSTEPPS^®^ strategies consistently. Overall responses suggested that high acuity within the MICU environment made engaging in TeamSTEPPS^®^ strategies difficult. An APP recalled an instance where acuity made engaging in appropriate roles difficult. “We went into a code situation and my colleague was assuming the team leader and then a different person, a resident, came in and started taking over and without a clear respect for the roles saying ‘this is my patient’”. An MD elaborated by explaining that TeamSTEPPS^®^ requires time to pause and discuss, but that can be difficult to maintain when there is high acuity. “Occasionally it’s hard to even do that if you can’t find the team because they’re going crazy because a couple of patients are crashing at the same time. And you have only so many people so you can’t say let’s stop and pause”. Even though TeamSTEPPS^®^ was developed to aid in high-stress situations, the healthcare team seemed to indicate that acuity made it difficult to follow TeamSTEPPS^®^ protocol.

## 4. Discussion

The selection of TeamSTEPPS^®^ training in the MICU was logical considering the literature supporting the benefits of teamwork training [4]. Implementation included education, feedback, reinforcement, and encouragement of teamwork behaviors among the interprofessional staff and trainees. While the COVID-19 pandemic interrupted our original plan to provide ongoing reinforcement of teamwork skills, it became a natural experiment and afforded the opportunity to show that teamwork training was utilized in the crisis and that skills were retained over time. Currently, to our knowledge, this is the first qualitative analysis showing the retention of TeamSTEPPS^®^ principles during a crisis. Previous research has called for studies on the retention of TeamSTEPPS^®^ training [17].

Utilization of TeamSTEPPS^®^ principles promoted teamwork through a shared mental model that provided individual team members with a clear understanding of their roles. Teams where everyone knows their role make fewer errors, thereby influencing patient safety [18]. This form of enhanced teamwork may help address issues around the interprofessional dynamics that are prevalent in healthcare organizations and can contribute to medical error [19]. Moreover, our interprofessional participants reported a consensus of feeling valued and respected, empathic towards each other, as well as trusted and trusting. These findings suggest an emotional element to teamwork that is not often reported. The significant number of comments on the emotional impact that teamwork had on participants of this study suggests a benefit for the individual team members, which is important for the collaboration necessary in teamwork, but rarely mentioned as an indirect benefit of TeamSTEPPS^®^. Teamwork in healthcare requires harmony and the willingness of individuals to cooperate and organize efforts towards a shared objective of patient care, and ultimately greater patient safety [20]. TeamSTEPPS^®^ training may have empowered individuals in their own roles as well as supported cohesion within the team.

Positive relationships between members of a healthcare team support respectful communication. As evidenced in the findings, there was consensus on the utility of TeamSTEPPS^®^ to enhance communication before, during, after, and between care events leading to improved patient safety. This finding is supported by previous research that credits the implementation of TeamSTEPPS^®^ strategies to workplace civility [21]. Communication is the backbone of an effective team. The complexity of the healthcare environment may necessitate the standardization of a protocol for communication [22], which TeamSTEPPS^®^ provided. Specifically, situation monitoring enabled team members to monitor performance and communicate progress in order to adapt to situations as they arise. Ingrained in TeamSTEPPS^®^ principles, engaging in briefings and debriefings has been associated with an increased ability to innovate in the PICU and SICU environment [23].

The MICU presented a unique environment for studying the use and sustainability of TeamSTEPPS^®^ given the high acuity present. The training and implementation of TeamSTEPPS^®^ strategies withstood the challenges of the COVID-19 crisis in the MICU, lending evidence to the retention of the teamwork strategies, despite the cessation of the reinforcement stage of training and the other barriers impacting overall team functioning and communication. This may be encouraging considering recent publications that suggest a decline in teamwork during COVID-19 at select health systems across the US [24]. Surprisingly, TeamSTEPPS^®^ did not uniformly enhance team functioning during the COVID-19 surge. Can we learn even more from the military about the use of team strategies during a crisis and conditions like the 2020 COVID-19 surge? A recent qualitative military study revealed that teamwork is enhanced when deployment occurs in extreme environments versus deployment in less extreme circumstances [25]. Perhaps with longer TeamSTEPPS^®^ reinforcement phases, as suggested by participants, the MICU personnel would have more uniformly viewed the brief “stop and pause” of teamwork as a facilitator versus a barrier to patient care in crisis.

Limitations of this study include this being a single unit study, interruption of training reinforcement due to a pandemic, and the retention of staff. We found that many of our ICU clinicians, particularly nurses, had left our institution in the time period from training to focus groups. We were unable to determine if any principles had been retained in that subgroup. Additionally, like other work in the literature that has thus far failed to demonstrate strong evidence of effectiveness in patient safety terms [26], this study was not designed to evaluate such results of the TeamSTEPPS^®^ training. A metanalysis utilizing the Kirkpatrick model (a globally recognized model of evaluating training programs) to assess crew resource management training formats in healthcare revealed that only a third of studies reported data on direct results (Kirkpatrick level 4) such as patient outcomes; however, in the studies that did report level 4 data, the changes in intervention groups were no different than the control groups [2]. This suggests a need for a more systematic evaluation of teamwork training modalities.

We encourage other MICUs to incorporate TeamSTEPPS^®^ training for teams as a part of their onboarding process, and the impact of this training be studied. We understand that other units may not have the resources for observation, feedback, debriefing, and reinforcement, but our findings suggest that even minimal TeamSTEPPS training may prove helpful. Future research should focus on the value of these resources for the MICU. Critical care management may benefit from incorporating the lessons learned from our endeavor.

## 5. Conclusions

Our themes from the focus group reveal how the training and implementation of TeamSTEPPS^®^ strategies withstood the challenges of the COVID-19 crisis in the MICU and overall positively impacted team functioning and communication.

## Data Availability

Not applicable.

## Appendix A. Focus Group Interview

### Introduction

In the Summer/Fall months of 2019, you participated in teamwork training (TeamSTEPPS^®^-team strategies to enhance performance and patient safety), either through a seminar delivered by Dr. Carol A. Terregino, senior associate dean for education and a medical student veteran (Jared Escobar M3) or via individualized training about TeamSTEPPS^®^ by Dr. Sugeet Jagpal (Quality and Safety MICU) or Dr. Payal Parikh (vice chair quality and safety, Department of Medicine). You should respond to these questions using the time frame of the academic year 2019–2020, after you received your training about TeamSTEPPS^®^.

1. What did you find to be the most memorable part of that team training?
2. Think about a memorable time you consciously used or observed the use of teamwork training in your daily activities. Tell the story and give your impressions about the event.
3. What did you notice about leadership and team setup during August 2019–June 2020?
4. What did you notice about the MICU briefs and debriefs?
5. What did you notice about the interactions between health professionals?
6. What did you notice about situation monitoring in the MICU?
7. What did you notice about mutual support in the MICU?
8. Think about a MICU experience where the team was high performing; what were the characteristics of the team?
9. What barriers to team performance have you identified?
10. What are changes you have noticed about team functioning in the MICU?
11. What did you notice about how teams functioned during the initial COVID-19 surge?
12. What have you noticed about interprofessional collaboration?
13. What have you noticed about running urgent/emergent situations, or codes?
14. What have you noticed about your personal practice efficiency?
15. In what role did you perceive Drs. Jagpal and Parikh to serve as Learning Health System Champions?
16. Think about a memorable time you consciously used or observed the use of TeamSTEPPS^®^ in your daily activities. Tell the story and give your impressions about the event.
17. What are your thoughts on providing this type of training in other units of the hospital?

## Appendix B. Codebook

**Code**	**Theme**	**Quick Definition**
	**How training impacted teamwork**	**Change in culture of interprofessional teamwork for care of patients**
Whole	New meaning of “whole” team/Team Formation/Validation/Acknowledgement	Broadened definition of team and who should be included in rounds. Teams are formed at the beginning of shift. Validating and crediting all members of the team.
Role	Roles Designated	Importance of everyone knowing their role so they can meaningfully contribute
Trust	Trust	Increased trust amongst the team/rapport/respect
MM	Everyone is on the same page/Shared mental model	Making sure that the whole team is on the same page. Common goal of patient care.
C/E	Consistency/Efficiency	Consistency in how things are done. Becoming efficient in patient care
Empathy	Empathy	Increased empathy for team members
	**How training impacted communication**	**Improved communication and raised awareness of communication issues**
B/D	Briefing/Debriefing	Getting the team ready for the day or debriefing at end of day
SM	Situation Monitoring	Checking on progress to goals, on team members, and the environment
ToC	Communication earlier/consistently/Transitions of care	Sign-outs that were comprehensive regarding events and care priorities
Safety	Realizing importance of constant and consistent communication for patient safety	Realizing importance of communication as a result of TeamSTEPPS training compared to how it used to be especially for patient safety
Units	Importance of communication on/between hospital units	Use of TeamSTEPPS communication tools on and between hospital units
	**Factors that influenced the use of TeamSTEPPS**	**Factors that supported or inhibited the use of TeamSTEPPS strategies**
TrainP	Initial training or any reinforcement of the training-**positive**	Any **positive** comments about the initial training, reinforcement of training
TrainN	Initial training or any reinforcement of the training-**negative**	Any **negative** comments about the initial training, lack of reinforcement of training.
COVIDP	COVID EFFECTs-Positive	The **positive** effect on teamwork function because of COVID
COVIDN	COVID EFFECTs-Negative	The **negative** effect on teamwork function because of COVID
MICUN	The MICU Environment-Negative	How the MICU environment can **challenge** team functions
MICUP	The MICU Environment-Positive	How the MICU structure can **enhance** team functioning
Acuity	Acuity	Referring to the critical status of patients
Hierarchy	Hierarchy	Power dynamics leading to fear of frank expression
